# Heterosubtypic Protection Induced by a Live Attenuated Influenza Virus Vaccine Expressing Galactose-α-1,3-Galactose Epitopes in Infected Cells

**DOI:** 10.1128/mBio.00027-20

**Published:** 2020-03-03

**Authors:** Li-Meng Yan, Sylvia P. N. Lau, Chek Meng Poh, Vera S. F. Chan, Michael C. W. Chan, Malik Peiris, Leo L. M. Poon

**Affiliations:** a School of Public Health, Li Ka Shing Faculty of Medicine, The University of Hong Kong, Hong Kong; b Department of Medicine, Li Ka Shing Faculty of Medicine, The University of Hong Kong, Hong Kong; The Peter Doherty Institute for Infection and Immunity

**Keywords:** immunology, influenza, influenza virus vaccines, live vector vaccines, universal vaccine

## Abstract

Anti-galactose-α-1,3-galactose (anti-α-Gal) antibody is naturally expressed at a high level in humans. It constitutes about 1% of immunoglobulins found in human blood. Here, we designed a live attenuated influenza virus vaccine that can generate α-Gal epitopes in infected cells in order to facilitate opsonization of infected cells, thereby enhancing vaccine-induced immune responses. In the presence of normal human sera, cells infected with this mutant can enhance phagocytosis of human macrophages and cytotoxicity of NK cells *in vitro*. Using a knockout mouse strain that allows expression of anti-α-Gal antibody *in vivo*, we showed that this strategy can increase vaccine immunogenicity and the breadth of protection. This vaccine can induce 100% protection against a lethal heterosubtypic group 1 (H5) or group 2 (mouse-adapted H3) influenza virus challenge in the mouse model. In contrast, its heterosubtypic protective effect in wild-type or knockout mice that do not have anti-α-Gal antibody expression is only partial, demonstrating that the enhanced vaccine-induced protection requires anti-α-Gal antibody upon vaccination. Anti-α-Gal-expressing knockout mice immunized with this vaccine produce robust humoral and cell-mediated responses upon a lethal virus challenge. This vaccine can stimulate CD11b^lo/−^ pulmonary dendritic cells, which are known to be crucial for clearance of influenza virus. Our approach provides a novel strategy for developing next-generation influenza virus vaccines.

## Abstract (precis)

Influenza A viruses have multiple HA subtypes that are antigenically diverse. Classical influenza virus vaccines are subtype specific, and they cannot induce satisfactory heterosubtypic immunity against multiple influenza virus subtypes. Here, we developed a live attenuated H1N1 influenza virus vaccine that allows the expression of α-Gal epitopes by infected cells. Anti-α-Gal antibody is naturally produced by humans. In the presence of this antibody, human cells infected with this experimental vaccine virus can enhance several antibody-mediated immune responses *in vitro*. Importantly, mice expressing anti-α-Gal antibody *in vivo* can be fully protected by this H1N1 vaccine against a lethal H5 or H3 virus challenge. Our work demonstrates a new strategy for using a single influenza virus strain to induce broadly cross-reactive immune responses against different influenza virus subtypes.

## INTRODUCTION

Influenza is a highly contagious disease associated with a major impact on global public health (1). There are 16 HA and 9 NA subtypes identified in avian populations. These HA subtypes can be classified into two groups (group 1: H1, H2, H5, H6, H8, H9, H11, H12, and H16; group 2: H3, H4, H7, H14, and H15). Of these subtypes, only H1N1, H2N2, and H3N2 viruses are known to cause pandemics. The secondary attack rates of seasonal and pandemic influenza in humans are estimated to be about 15% and 33%, respectively (2). Given the fact that animal influenza viruses may have the potential to trigger pandemics, there is an urgent need for effective and “universal” strategies to control influenza. Of the existing control measures, vaccination is one of the most effective ways to prevent influenza virus infections. Currently, there are two major classes of influenza virus vaccines commercially available for human use, i.e., inactivated and live attenuated vaccines (3, 4). Both types of vaccines have their advantages and disadvantages. However, none of the currently available vaccines are designed for inducing broadly cross-protective immune responses against different animal influenza virus subtypes, a key feature of universal influenza virus vaccines. The development of novel strategies leading to an effective and safe universal influenza virus vaccine remains a grand challenge.

Antigen uptake by professional antigen-presenting cells (APCs) such as dendritic cells (DCs) plays a key role in adaptive immunity. The antigen uptake process can be enhanced by opsonization of antigens, and a high level of pre-existing antibodies specific for the antigen can facilitate this process (5). Recent findings indicate that the expression of galactose-α-1,3-galactose (α-Gal) epitopes on membrane surfaces can enhance their opsonization, leading to phagocytosis by APCs mediated by anti-Gal antibodies, which are naturally expressed in healthy individuals. This results in enhanced phagocytosis of infected cells by professional APCs (6). Human polyclonal anti-α-Gal antibodies exist in different forms (IgA, IgG, and IgM), and they are abundantly expressed in healthy individuals (∼1% of circulating immunoglobulins). α-Gal epitopes are expressed by a wild range of living organisms. This epitope is presented by surface glycoproteins or glycolipids after the enzymatic reaction of α-1,3-galactosyltransferase (α-1,3-GT) within the endoplasmic reticulum (6). However, humans, apes, and Old World monkeys have a defective α-1,3-GT gene, and they are unable to express this epitope (6). Because of the continuous stimulation with α-Gal epitopes in foods and normal bacteria flora, a high level of anti-α-Gal antibodies is naturally produced by these primates throughout their lifetime.

The use of the α-Gal epitope to stimulate T and B cell responses through enhancing antigen uptake has been demonstrated in cancer therapy and experimental vaccines (7–9). Previous viral vaccine studies were based on inactivated viral antigens artificially coated with α-Gal epitopes, and these vaccines were shown to enhance the adaptive immune response against a homologous virus infection. However, this strategy requires additional enzymatic processing to generate α-Gal epitopes on these antigens, thereby reducing antigen yields and cost-effectiveness. We have therefore designed an attenuated influenza virus that contains an α-1,3-GT gene in order to express α-Gal epitopes in infected cells. In addition, we also reason that the expression of viral RNA and viral proteins in infected cells upon vaccination can further enhance vaccine-induced immune responses. Our results demonstrate that this approach is a promising vaccine strategy to induce heterosubtypic protection against influenza.

## RESULTS

### Characterization of an NA mutant expressing α-1,3-galactosyltransferase *in vitro*.

**FIG 1:**
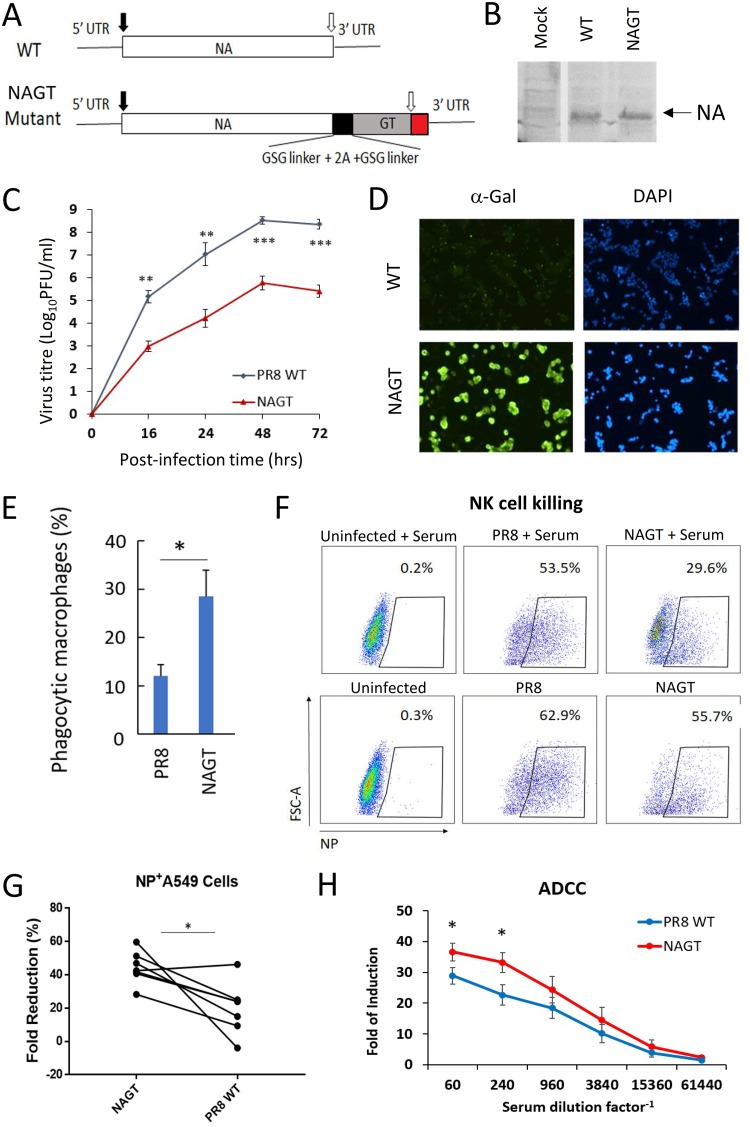
Characterization of the NAGT mutant *in vitro*. (A) WT and mutated NA segments in the cRNA sense strand. A mouse α-1,3-GT gene ORF was introduced immediately before the stop codon of the NA ORF (open arrow). An autoproteolytic cleavage site of porcine teschovirus-1 flanked by two short peptide linkers (GSG) was introduced into the junction of NA and α-1,3-GT ORF as shown. 5′ and 3′ untranslated regions (UTRs) and the start codon (black arrow) are indicated. The red box represents the 5′-end vRNA packaging signal sequence at the NA ORF region. (B) Detection of influenza virus NA protein infection by Western blotting. Human A549 cells infected with WT and NAGT viruses (MOI, 1) were harvested at 48 h postinfection. (C) Replication kinetics of NAGT mutant in MDCK cells (MOI, 0.001). Progeny virus titers were determined using standard plaque assays. (D) Immunofluorescent staining of α-Gal epitopes in human A549 cells infected with WT or NAGT virus at 24 h postinfection (MOI, 1). Signals from the nuclear counterstain (DAPI) are also shown. (E) Human A549 cells infected with the NAGT virus can enhance phagocytic activities of human monocyte-derived macrophages. (F) Antibody-dependent NK cell assay. Human A549 cells treated with PBS (left), WT virus (middle), or the NAGT virus (right) were incubated with activated human NK cells in the presence (top) or absence (bottom) of heat-inactivated human serum. The percentages of influenza virus NP-positive cells after incubation under different experimental conditions are shown. These images show representative fluorescence-activated cell sorting (FACS) plots obtained by using a serum sample from a healthy donor. (G) Stimulating effect of all tested human sera on NK cells for killing WT- and NAGT-infected A549 cells (fold reduction in NP^+^ A549 cells; *n* = 6; paired *t* test). (H) Luciferase reporter assay for ADCC activity. A549 cells infected with the WT or NAGT virus were first treated with serially diluted human serum samples and then tested by ADCC assays. Each data point represents the average reading from six different human serum samples; values are means ± standard deviations (SD). *, *P* < 0.05; **, *P* < 0.01; ***, *P* < 0.001.

To express α-Gal epitopes in infected cells, a recombinant A/PR/8/34 H1N1 (PR8) virus carrying a mouse α-1,3-GT gene in its neuraminidase (NA) viral RNA (vRNA) segment was made (Fig. 1A); we called this the NAGT mutant. The α-1,3-GT gene was inserted immediately before the stop codon of the NA open reading frame (ORF) in the same sense. A 2A peptide derived from porcine teschovirus-1 flanked by short peptide linkers (GSG) was inserted into the junction of NA and α-1,3-GT ORFs in order to allow an efficient cleavage of NA–α-1,3-GT polypeptide into NA and α-1,3-GT proteins (Fig. 1B) (10). The packing signal at the 5′ end of the NA vRNA (the untranslated region and 157 nt of the ORF region) was inserted next to the stop codon of the NA-GT ORF sequence. The NAGT mutant was attenuated in MDCK cells (∼2.5-log reduction in virus titer), but it was still able to achieve a relatively robust replication in infected cells (maximum titer, ∼5.8 × 10^5^ PFU/ml) (Fig. 1C). Our preliminary mouse infection studies showed that the NAGT mutant is attenuated in mice (50% mouse-lethal dose [MLD_50_] of the wild type [WT] = 178 PFU; MLD_50_ of the NAGT mutant = 2,700 PFU). We also confirmed that human cells infected with the NAGT mutant, but not the WT virus, could express α-Gal epitopes both in cytoplasm and on cell surfaces (Fig. 1D; also, see Fig. S1 in the supplemental material).

10.1128/mBio.00027-20.1 FIG S1 **Detection of α-Gal epitopes on nonpermeabilized cells.** Human A549 cells treated with the NAGT mutant and PBS (control) were stained for α-Gal epitopes. The staining protocol is identical to the one used for Fig. 1D, except that Triton X-100 was not used in the initial cell fixation. DAPI was used as a nuclear counterstain. Download FIG S1, PDF file, 0.4 MB. Copyright © 2020 Yan et al. 2020 Yan et al. This content is distributed under the terms of the Creative Commons Attribution 4.0 International license.

We hypothesized that the expression of α-Gal epitopes by the NAGT mutant can lead to binding of human anti-α-Gal antibody to infected cells, enhancing opsonization of infected cells by APCs and thereby boosting vaccine-induced adaptive responses. To test this hypothesis, cells infected with WT and NAGT viruses were treated with normal human sera, and we assessed the ability of primary human monocyte-derived macrophages to engulf these cells. As shown in Fig. 1E, human A549 cells infected with NAGT mutants could significantly enhance the phagocytic activity of human macrophages (*P* < 0.05). Cells infected with the NAGT mutant were more susceptible to killing by NK cells after incubation with human sera (Fig. 1F, right). This enhanced killing effect was much less pronounced in cells infected with WT virus (Fig. 1F, middle). The lower degree of killing effect stimulated by the WT virus-infected cells could be attributed to the presence of influenza virus antibodies commonly found in normal individuals. Cells infected with the NAGT mutant in 6 human serum samples were more likely to be killed by NK cells than those infected with WT virus in 5 samples (Fig. 1G) (*P* < 0.05). We further confirmed the stimulating effect of the NAGT mutant on immune effector cells by using an antibody-dependent cellular cytotoxicity (ADCC) reporter assay (Fig. 1H). Overall, the above results indicated that the expression of α-Gal epitopes by NAGT-infected cells can stimulate phagocytosis of APCs, NK cell-mediated cytotoxicity, and ADCC.

### Characterization of α-1,3-GT knockout mice after vaccination with the NAGT mutant.

**FIG 2:**
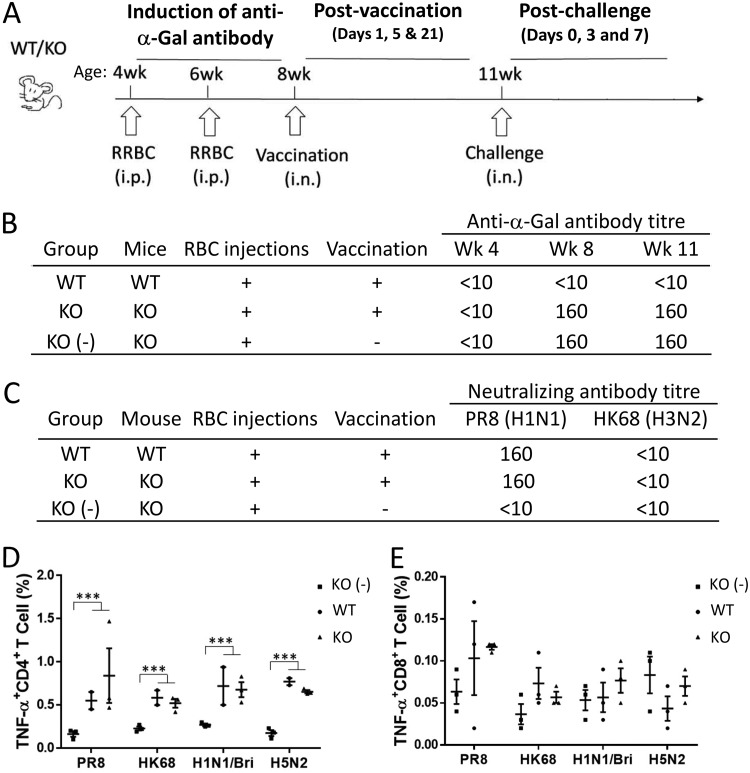
Vaccination with the NAGT mutant in WT and α-1,3-GT KO mice. (A) General scheme of vaccination and virus challenge. Mice were primed with rabbit RBCs and given boosters via the intraperitoneal route at 4 and 6 weeks of age. Mice were vaccinated with the NAGT mutant at 8 weeks of age and then challenged with a lethal dose of influenza virus via the intranasal route. Relevant samples were collected at 4, 6, and 8 weeks of age and at various postvaccination (days 1, 5, and 11) and postchallenge (days 0, 3, and 7) time points. (B) Anti-α-Gal antibody titers in different experimental groups before virus challenge in week 11. (C) H1N1 and H3N2 virus-specific neutralizing antibody titers in different experimental groups before virus challenge in week 11. (D and E) Splenic CD4^+^ and CD8^+^ T-cell recall responses against different influenza viruses (H1N1/PR8, H3N2/HK68, H1N1/Brisbane/07, and H5N2/HK/MPF461/07). T cells were studied with ICS assays. Percentages of activated T cells (TNF-α^+^) are shown. Data are means ± SD. ***, *P* < 0.001.

WT laboratory mice have a functional α-1,3-GT gene, and they do not produce anti-α-Gal antibodies, thus avoiding autoimmunity. An α-1,3-GT knockout (KO) mouse strain was therefore used to evaluate the vaccine potential of the NAGT mutant (Fig. 2A) (11). These KO mice were first intraperitoneally injected with rabbit red blood cells (RBCs) twice to stimulate anti-α-Gal antibody production (12). A high level of anti-α-Gal antibody could be stably produced and maintained by the RBC-treated KO mice (weeks 8 to 11) (Fig. 2B). In contrast, no anti-α-Gal antibody could be detected in RBC-treated WT mice.

With a predetermined intranasal vaccination dose (150 PFU), both WT and KO vaccinated mice produced high titers of neutralizing antibody against H1N1, but not H3N2, virus at 3 weeks postvaccination (Fig. 2C), indicating that the vaccine virus by itself did not stimulate heterosubtypic neutralizing antibody production. Influenza virus-specific CD4^+^ or CD8^+^ T-cell recall responses (tumor necrosis factor alpha positive [TNF-α^+^]) in spleen tissues of vaccinated mice against different influenza virus subtypes were measured *in vitro* using intracellular cytokine staining (ICS) assays. In comparison to the results from mock-vaccinated KO mice, splenic CD4^+^ T cells from both vaccinated WT and KO mice could be stimulated by different viral subtypes (H1N1, H3N2, and H5N2; *P* < 0.05) (Fig. 2D), indicating that there were cross-reactive CD4^+^ T-cell responses in vaccinated mice. Cross-reactive CD8^+^ T-cell responses could not be observed in vaccinated mice, although both vaccinated WT and KO mice have some, although insignificant, CD8^+^ recall responses to the homologous virus (PR8) (Fig. 2E). Hence, the antibody and T-cell responses of vaccinated WT and KO mice at day 21 postvaccination were similar.

**FIG 3:**
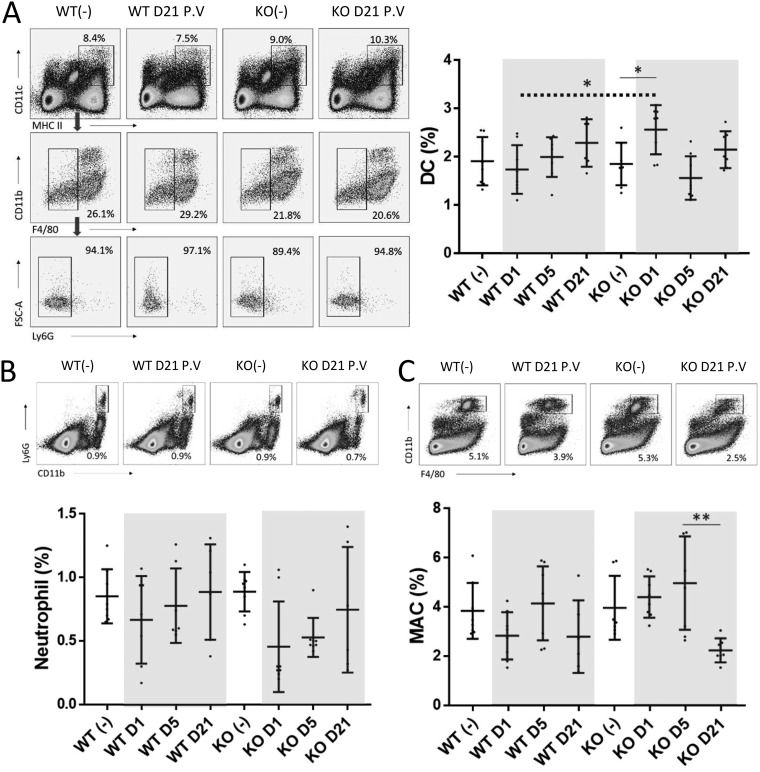
Professional antigen-presenting cells (DCs and macrophages) and neutrophils in spleen tissue after vaccination. Spleens from vaccinated WT and KO mice were harvested at days 1, 5, and 21 postvaccination. Unvaccinated mice (−) were used as controls. Representative FACS plots and percentages of DCs (A), neutrophils (B), and macrophages (C) in all studied samples are shown. Data from vaccinated mice are highlighted. The dotted line shows the comparison between KO and WT mice at the same time point (*t* test). Data are means ± SD. *, *P* < 0.05; **, *P* < 0.01.

**FIG 4:**
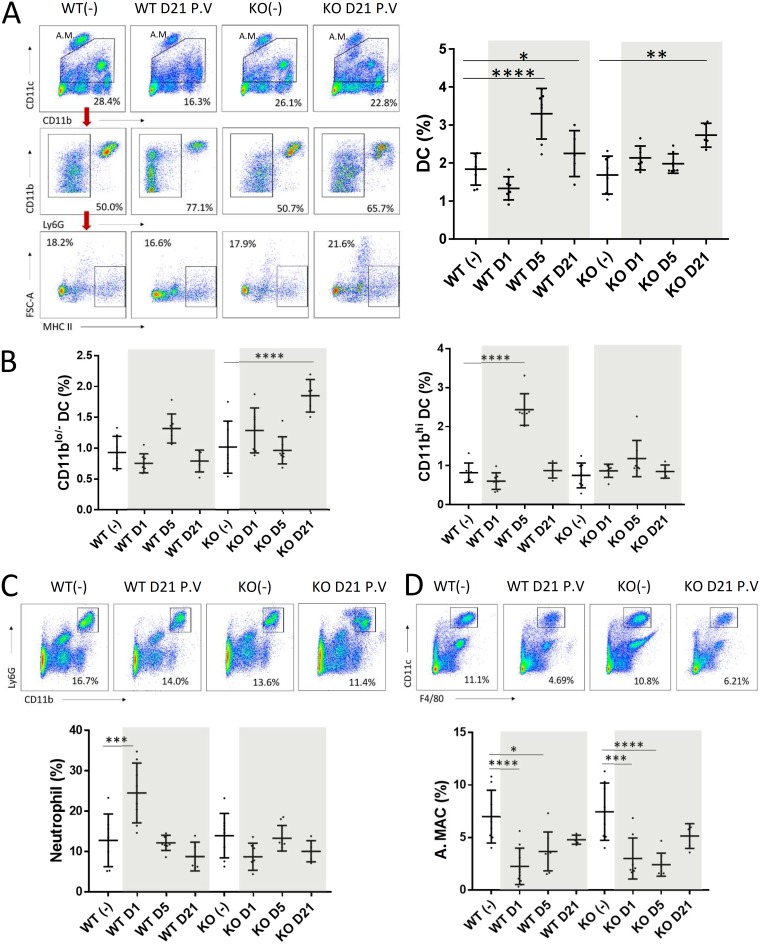
Professional antigen-presenting cells (DCs and alveolar macrophages) and neutrophils in lung tissue after vaccination. Lungs from vaccinated WT and KO mice were harvested at days 1, 5, and 21 postvaccination. Unvaccinated mice (-) were used as controls. Representative FACS plots and percentages of DCs (A), CD11b^lo/−^ and CD11b^+^ DCs (B), neutrophils (C), and alveolar macrophages (D) in all studied samples are shown. A.M., alveolar macrophages (38). Data from vaccinated mice are highlighted. Data are means ± SD. *, *P* < 0.05; **, *P* < 0.01; ***, *P* < 0.001; ****, *P* < 0.0001.

To determine whether the NAGT mutant can induce more robust responses in other immune cells, the frequencies of DCs, macrophages, and neutrophils in the spleens (Fig. 3) and lungs (Fig. 4) of vaccinated mice were studied at days 1, 5, and 21 postvaccination. An elevation of the DC population (CD11c^+^ MHC-II^hi^ Ly6G^−^ F4/80^−^) was observed in the spleen tissues of KO mice at day 1 postvaccination (Fig. 3A), indicating that this mutant can stimulate the migration of DCs into the spleen. In contrast, the neutrophil and macrophage profiles in the spleens of these WT and KO mice at these time points were similar (Fig. 3B and C).

An increased frequency of pulmonary DCs (CD11c^+^ MHC-II^hi^ Ly6G^−^ F4/80^−^) in these vaccinated mice was observed, peaking at day 5 and day 21 in vaccinated WT and KO mice, respectively (Fig. 4A). Interestingly, apart from the difference in kinetics, the studied DC populations in the WT and KO mice at these time points were slightly different (Fig. 4B; CD11b^hi^ versus CD11b^lo/−^). The increased DC population of vaccinated KO mice was primarily CD11b^lo/−^ (Fig. 4B, left). In contrast, a substantial increase in the CD11b^hi^ DC population (Fig. 4B, right) and a marginal increase in the CD11b^lo/−^ population (Fig. 4B, left; insignificant relative to the unvaccinated control) could be detected in vaccinated WT mice at day 5 postvaccination. An elevated neutrophil frequency in vaccinated WT mice at day 1 postvaccination was observed (Fig. 4C), suggesting that the NAGT mutant caused more severe inflammatory responses in WT mice because of the lack of anti-α-Gal antibody in these mice to control the infection (13). Both vaccinated WT and KO mice had reduced levels of alveolar macrophages after vaccination (Fig. 4D), but the reductions were indistinguishable.

Overall, these results indicated that the NAGT mutant can direct DC and other APC responses in WT and KO mice after vaccination.

### The NAGT mutant can protect mice against a lethal homologous challenge.

**FIG 5:**
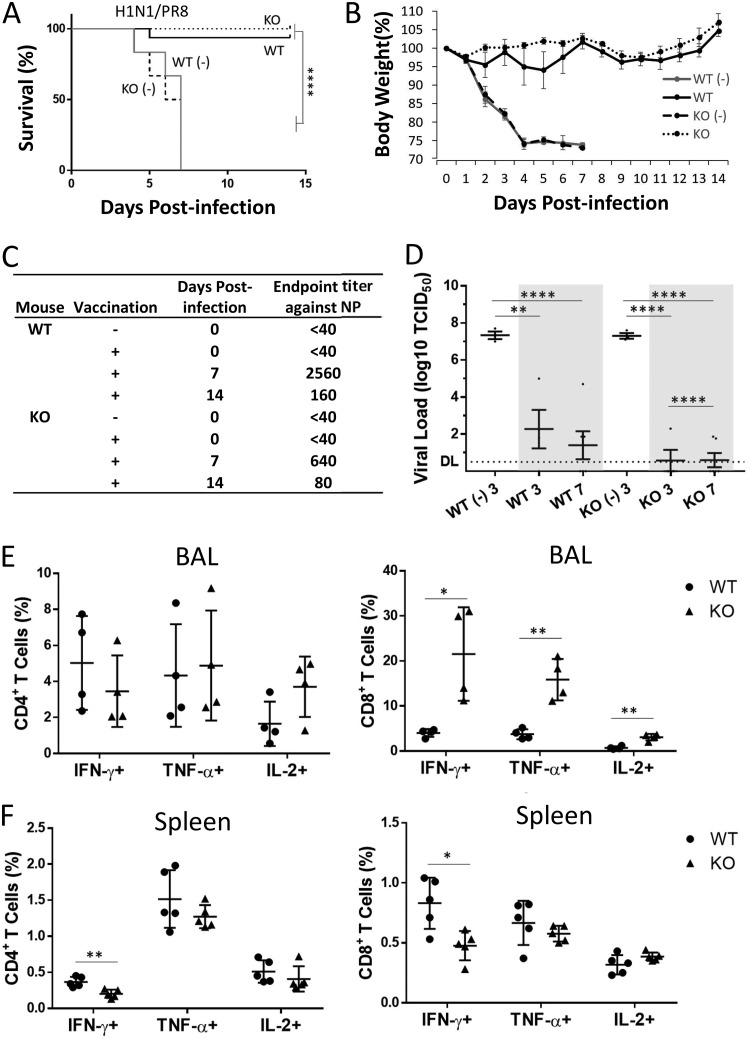
The NAGT mutant protects mice against a lethal homologous challenge. Vaccinated and unvaccinated (-) mice were challenged with a lethal dose of H1N1 (A/PR/8/34; 40 MLD_50_s) at 3 weeks postvaccination. (A) Survival rates were assessed daily for 14 days after challenge (log rank test). Each vaccinated group had 16 mice. (B) Weight loss in different mouse groups. (C) NP-specific antibody titers in mice at days 0, 7, and 14 after virus challenge. Antibody levels were studied by ELISA. (D) Lung virus titers in mice at days 3 and 7 postchallenge. Data from vaccinated mice are highlighted. DL, detection limit. (E and F) PR8-specific CD4^+^ and CD8^+^ T-cell recall responses in BAL fluid and spleens of infected mice at day 7 postinfection. Activities were determined by ICS assays. Data are means ± SD. *, *P* < 0.05; **, *P* < 0.01; ****, *P* < 0.0001.

The vaccine potential of NAGT mutant was first tested by a homologous challenge. Rabbit RBC-primed WT and KO mice were challenged by a lethal dose of WT PR8 virus (10 MLD_50_s) at day 21 postvaccination. All mock-vaccinated WT and KO mice died from the infection as expected. All and 94% of vaccinated KO and WT mice, respectively, survived the challenge (Fig. 5A), with no apparent weight loss in the vaccinated KO mouse group (Fig. 5B). Both vaccinated groups mounted good antibody responses against influenza virus NP protein after challenge (Fig. 5C). Although both vaccinated groups had greatly reduced virus lung titers compared to the unvaccinated control groups, vaccinated KO mice had a higher virus clearance rate than vaccinated WT mice (Fig. 5D).

Vaccine-induced influenza virus-specific T-cell responses in the respiratory (bronchoalveolar lavage [BAL] fluid) and spleen samples of treated mice were measured at day 7 postchallenge. Influenza virus-specific CD4^+^ and CD8^+^ T-cell responses were measured by gamma interferon (IFN-γ) ICS assays after an *ex vivo* stimulation with PR8. In BAL fluid, CD8^+^ T-cell responses of KO mice were much higher than those of WT mice (e.g., IFN-γ^+^ and TNF-α^+^) (Fig. 5E, right). In spleen samples, both IFN-γ^+^ CD4^+^ and CD8^+^ T-cell responses from WT mice were only slightly, yet significantly, better than those of KO mice (Fig. 5F). The profiles of DCs, neutrophils, and macrophages in lung and spleen tissues were also determined at days 0, 3, and 7 postchallenge (see Fig. S2), but no significant difference between these mice was detected.

10.1128/mBio.00027-20.2 FIG S2 **The NAGT mutant protects mice from a lethal homologous virus challenge**. Three weeks after vaccination, mice were challenged intranasally with a lethal dose of H1N1 virus (PR8; 10 MLD_50_s). (A) Percentages of total dendritic cells, neutrophils, and alveolar macrophages in lung tissues (left to right). (B) Percentages of total dendritic cells, neutrophils, and macrophages in spleen tissues (left to right). Data are means ± SD. Download FIG S2, PDF file, 0.7 MB. Copyright © 2020 Yan et al. 2020 Yan et al. This content is distributed under the terms of the Creative Commons Attribution 4.0 International license.

In short, the rapid virus clearance and the highly robust CD8^+^ T-cell responses observed in the vaccinated KO mice suggest that the NAGT mutant, in the presence of anti-Gal antibody, can induce enhanced immune responses in this mouse model.

### The NAGT mutant can protect mice against a lethal heterosubtypic H3N2 virus challenge.

**FIG 6:**
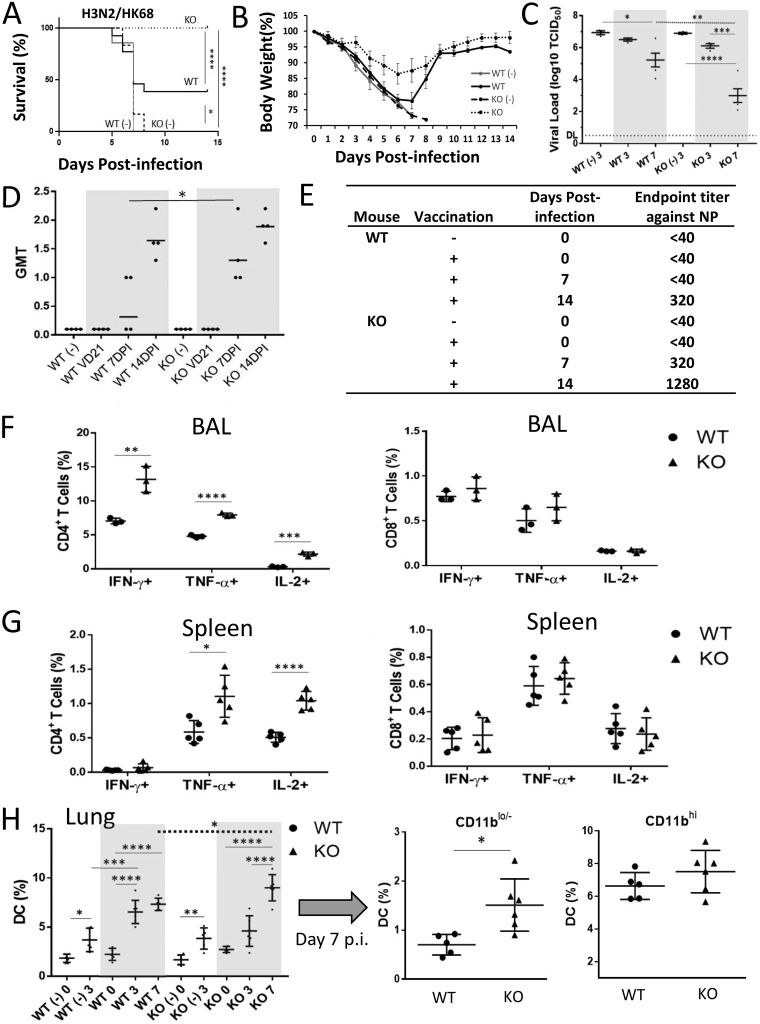
The NAGT mutant protects mice against a lethal heterosubtypic H3N2 challenge. Vaccinated and unvaccinated (-) mice were challenged with a lethal dose of H3N2 virus (HK68; 10 MLD_50_s) at 3 weeks postvaccination. (A) Survival rates were assessed daily for 14 days after challenge (log rank test). Each vaccinated group had ≥20 mice. (B) Weight loss in different mouse groups. (C) Lung virus titers in mice at days 3 and 7 postchallenge. Data from vaccinated mice are highlighted. DL, detection limit. (D) HK68-specific neutralizing antibody titers in mice before (3 weeks postvaccination) and after (days 7 and 14 postchallenge) challenge. Data from vaccinated mice are highlighted. (E) NP-specific antibody titers in mice at days 0, 7, and 14 after virus challenge. Antibody levels were studied by ELISA. (F and G) HK68-specific CD4^+^ and CD8^+^ T-cell recall responses in BAL fluid (F) and spleen tissue (G) of infected mice at day 7 postinfection. (H) Percentages of total DCs (left) and of CD11b^lo/−^ and CD11b^hi^ DCs (right) in infected lung tissues at day 7 postchallenge. The dotted line shows the comparison between KO and WT mice at the same time point (*t* test). Data are means ± SD. *, *P* < 0.05; **, *P* < 0.01; ***, *P* < 0.001; ****, *P* < 0.0001.

To determine the protective effect of the NAGT mutant against heterosubtypic infection, a mouse-adapted A/HK/1/68 H3N2 (HK68; 10 MLD_50_s) virus was used in the virus challenge. All vaccinated KO mice survived, whereas 38% of vaccinated WT mice and all unvaccinated mice died from the infection (Fig. 6A). In addition, vaccinated KO mice had less weight loss (Fig. 6B) and faster virus clearance in lungs (Fig. 6C) than the controls. Robust H3N2 virus-specific neutralizing antibody and NP-specific antibody responses could be detected in vaccinated KO mice, but not in vaccinated WT mice, at day 7 postchallenge (Fig. 6D and E). Furthermore, surviving vaccinated WT mice had much less NP-specific antibody than vaccinated KO mice at day 14 postchallenge. These data indicated that the NAGT mutant can induce potent heterosubtypic antibody responses in KO mice against the H3N2 virus. Such enhanced heterosubtypic protection was confirmed to require the presence of anti-α-Gal antibody in KO mice (see below).

To determine the mechanism of protection in vaccinated KO mice, CD4^+^ and CD8^+^ T-cell responses in vaccinated WT and KO mice at day 7 postchallenge were studied. H3N2 influenza virus-specific CD4^+^ T-cell responses were found to be much stronger in the lung and spleen tissues of vaccinated KO mice than those of vaccinated WT mice (Fig. 6F and G, left). In contrast, no difference was detected between the CD8^+^ T-cell responses of these two vaccinated groups (Fig. 6F and G, right).

The DC, neutrophil, and macrophage populations in infected lung and spleen tissues were studied. Lung tissues from both vaccinated groups had increased frequencies of DCs after challenge (Fig. 6H, left) and these were predominantly CD11b^hi^ DC responses (Fig. 6H, right, and data not shown). Interestingly, although the levels of CD11b^hi^ DCs in vaccinated WT and KO mice were similar, the CD11b^lo/−^ DC levels in vaccinated KO mice were significantly higher than those in vaccinated WT mice at day 7 postchallenge (Fig. 6H, right). In spleen samples, no significant increase in the DC population after challenge was observed in any vaccinated group (see Fig. S3). The neutrophil and macrophage profiles in infected WT and KO mice were also similar (see Fig. S3).

10.1128/mBio.00027-20.3 FIG S3 **The NAGT mutant protects mice from a lethal heterosubtypic H3N2 virus challenge.** Three weeks after vaccination, mice were challenged intranasally with 10 MLD_50_s of an H3N2 virus (HK68). (A) Percentages of neutrophils (left) and alveolar macrophages (right) in lung tissues. (B) Percentages of total dendritic cells, neutrophils, and macrophages in spleen tissues (left to right). Data are means ± SD. Download FIG S3, PDF file, 0.7 MB. Copyright © 2020 Yan et al. 2020 Yan et al. This content is distributed under the terms of the Creative Commons Attribution 4.0 International license.

### The NAGT mutant can protect mice against a lethal highly pathogenic H5N1 virus challenge.

**FIG 7:**
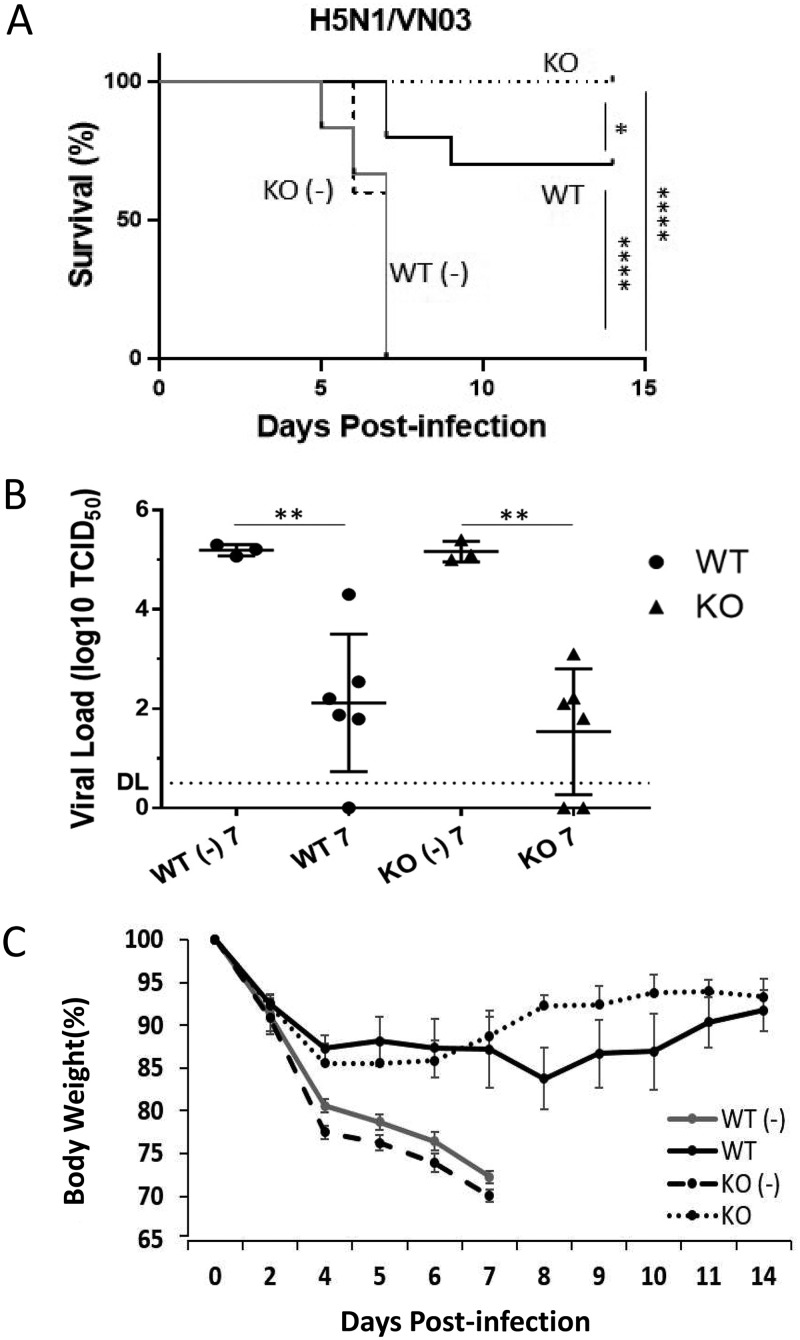
The NAGT mutant protects mice against challenge with a lethal heterosubtypic highly pathogenic H5N1 virus. Vaccinated and unvaccinated (-) mice were challenged with a lethal dose of H5N1 virus (VN1203; 10 MLD_50_s) at 3 weeks postvaccination. (A) Survival rates were assessed daily for 14 days after challenge (log rank test). Each vaccinated group had 10 mice. (B) Lung virus titers in mice at day 7 postchallenge. DL, detection limit. (C) Weight loss in different mouse groups. Data are means ± SD. *, *P* < 0.05; **, *P* < 0.01; ****, *P* < 0.0001.

The protective effect of the NAGT mutant against infection with the highly pathogenic H5N1 avian influenza virus was studied in the mouse model (A/VN/1203/04; 10 MLD_50_s). All vaccinated KO mice survived a lethal virus challenge (Fig. 7A), whereas vaccinated WT mice had a significantly reduced survival rate (70%; *P* < 0.05). The lung virus titers of these two vaccinated groups at day 7 postchallenge were not significantly different (Fig. 7B). However, a more rapid recovery was observed in the vaccinated KO group in week 2 postchallenge (Fig. 7C). Thus, these results, together with those from the work with H3N2 described above, indicate that the NAGT mutant can elicit strong, broadly cross-reactive immune responses against both group 1 and group 2 influenza virus infections.

### Enhanced heterosubtypic protection requires anti-α-Gal antibody in KO mice.

**FIG 8:**
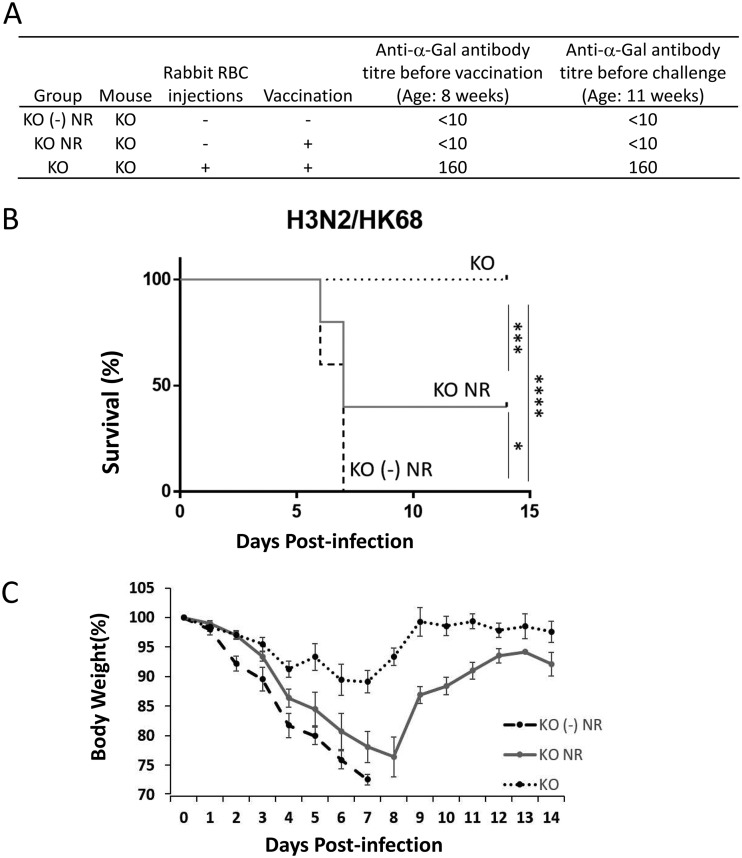
Enhanced protection by the NAGT mutant requires anti-α-Gal antibody in mice. (A) Anti-α-Gal antibody titers in mice immediately before vaccination and virus challenge. KO mice with (KO) or without (KO NR) rabbit RBC stimulation were vaccinated at 8 weeks of age. Mock-treated KO mice were used as controls [KO (-) NR]. Serum anti-α-Gal antibody titers in these mouse groups were determined by ELISA. (B). Survival rates of these mouse groups after a lethal HK68 virus challenge (10 MLD_50_s). Each group had 5 mice. (C) Weight loss in different mouse groups. Data are means ± SD. *, *P* < 0.05; ***, *P* < 0.001; ****, *P* < 0.0001.

To demonstrate that the above-described enhanced vaccine-induced protective effect in KO mice was due to the recognition of α-Gal epitopes by anti-α-Gal antibody, KO mice with or without prior rabbit RBC injections were vaccinated and challenged using the protocol used for the H3 model. Mice without the RBC treatment did not have detectable levels of anti-α-Gal antibody before vaccination and virus challenge (Fig. 8A). In the absence of anti-α-Gal antibody *in vivo*, the enhanced heterosubtypic protective effects of NAGT mutant disappeared (Fig. 8B, group KO NR). The survival rate of this vaccinated group (40%) was similar to the one observed in vaccinated WT mice (38%) (Fig. 6A). In addition, this group of mice had more severe weight loss than the fully protected group (Fig. 8C). These results demonstrate that, without prior rabbit RBC injections, the α-1,3-GT KO and WT mice have similar susceptibilities to influenza virus infection. These results also confirm that the enhanced protective effect of the NAGT mutant depends on the presence of anti-α-Gal antibody *in vivo*.

## DISCUSSION

In this study, we generated an attenuated influenza virus to express α-Gal epitopes in infected cells. Results from our *in vitro* human models indicated that, in the presence of anti-α-Gal antibody, NAGT virus-infected cells could enhance phagocytosis, NK cell-mediated cell killing, and ADCC. These enhanced effects suggested that the opsonization of infected cells by anti-α-Gal antibody can enhance vaccine-induced responses. Indeed, the enhanced protective effects of the vaccine were observed in a KO mouse model that expresses a high level of anti-α-Gal antibody *in vivo*. In this mouse model, the NAGT mutant induced broadly cross-reactive protection against both group 1 (H1 and H5) and group 2 (H3) influenza viruses. With only a single intranasal vaccination, vaccinated KO mice developed enhanced humoral and/or cell-mediated responses after virus challenge.

Our vaccinated mice did not have detectable pre-existing neutralizing antibody against H3N2 virus before challenge. Results from the H3 virus challenge model indicated that the vaccinated KO mice could develop prompt and robust antibody responses against both viral surface and internal proteins after the challenge (Fig. 6D and E). These suggested that NAGT-primed KO mice have effective cross-reactive memory responses to heterosubtypic influenza viruses, thereby activating an early humoral response to control the infection. The exact immune mechanisms that contribute to such rapid antibody responses are yet to be fully determined. With the enhanced T-cell responses observed in our mouse models, it is likely that the enhanced protection might involve interactions between T- and B-cell compartments.

In vaccinated KO mice, a homologous challenge triggered robust CD8^+^ T-cell recall responses (Fig. 5E), whereas a heterosubtypic challenge induced robust CD4^+^ T-cell recall responses (Fig. 6F). Many T cells in the BAL fluid of these mice are polyfunctional cytokine-producing T cells (see Fig. S4 in the supplemental material), indicating that vaccinated KO mice had better T-cell responses than vaccinated WT mice to control virus infection. These differential CD4^+^ and CD8^+^ responses in vaccinated KO mice also suggested that the protective mechanisms of the NAGT mutant in the studied H1 and H3 models are different. Specifically, there were enhanced CD4^+^ T-cell recall responses in the BAL fluid and spleen samples of vaccinated KO mice after a H3 virus challenge. We and others previously demonstrated that memory CD4^+^ T cells are crucial for heterosubtypic influenza virus protection (14, 15). Depleting memory CD4^+^ T cells in mice vaccinated with a vaccinia virus-vectored universal influenza virus vaccine can increase mortality, delay antibody production, and reduce CD8^+^ T-cell recall response after a heterosubtypic challenge (16). Our current findings from the H3 model are in line with these previous findings.

10.1128/mBio.00027-20.4 FIG S4 **Vaccination with the NAGT mutant can enhance polyfunctional T-cell responses in BAL fluid upon virus challenge.** (A) PR8-specific polyfunctional CD8^+^ T cells in BAL fluid at day 7 postchallenge. Data were reanalyzed from those presented in Fig. 5E (right). (B) HK68-specific polyfunctional CD4^+^ T cells in BAL fluid at day 7 postchallenge. Data were reanalyzed from those presented in Fig. 6F (left). Data are means ± SD. *, *P* < 0.05; **, *P* < 0.01; ***, *P* < 0.001. Download FIG S4, PDF file, 0.4 MB. Copyright © 2020 Yan et al. 2020 Yan et al. This content is distributed under the terms of the Creative Commons Attribution 4.0 International license.

DCs are vital for immune surveillance. This cell population can help to eliminate pathogens via adaptive immune responses. Opsonized immune complexes can be efficiently internalized by monocyte-derived DCs via interactions between the Fc portion of antibodies and Fcγ receptors of DCs (17). Antigen-loaded DCs can migrate to draining lymph nodes to activate T cells. Increasing such DC–T-cell interactions in lymphoid systems is expected to enhance immune memory responses against influenza virus (18, 19). Mouse lung DCs (CD11c^+^ MHC-II^hi^) can be divided into two major subpopulations: CD11b^lo/−^ and CD11b^hi^ DCs (20). In this study, both vaccine groups had increased total DC populations in lung tissues after vaccination, but the increased DC subsets in these vaccinated mice were different. The increased DC population in vaccinated KO mice was entirely CD11b^lo/−^, whereas the increased DC population in vaccinated WT mice was predominantly CD11b^hi^ (Fig. 4B). This suggests that the NAGT mutant can modulate protective DC responses in these mouse groups. Interestingly, an increased CD11b^lo/−^ DC frequency was also observed in vaccinated KO mice after a heterosubtypic challenge (Fig. 6C). Our gating strategy for DCs also suggests that these were classical DCs (cDCs). Nonlymphoid cDCs have two major subsets: CD103^+^ CD11b^−^ cDCs and CD103^−^ CD11b^+^ cDCs. Hence, the CD11b^lo/−^ DCs in this study were considered CD103^+^ cDCs (20–23). Previous studies demonstrated that CD11b^hi^ and CD11b^lo/−^ cDCs have distinct functions. Briefly, lung CD11b^hi^ cDCs are potent inducers of Th2 and Th17 responses against extracellular pathogens (24, 25), and CD103^+^ cDCs favor Th1 responses by priming CD4^+^ T cells and cross-presenting soluble and apoptosis cell-associated antigens to CD8^+^ T cells via major histocompatibility complex class I (MHC I) receptors (26, 27). In addition, CD11b^lo/−^ cDCs are known to play critical roles for influenza virus clearance (28). This cell population acquires influenza virus antigens through phagocytosis of infected cells, but not by infection, and it can facilitate the lung homing capacity of differentiated T cells to lymph nodes (28–30). The ability of NAGT to enhance CD11b^lo/−^ cDC responses might therefore be beneficial against influenza virus infection. Further characterization of this CD11b^lo/−^ cDC population might help to explain the NAGT mutant-induced heterosubtypic protection.

The NAGT mutant is about 15 times less virulent than its WT control in mice. Although this virus background (PR8) is lethal in mice, it is a standard master strain for making seasonal influenza virus vaccines. Nonetheless, further attenuation of the mutant might be needed. We previously reported the use of codon bias to fine-tune the level of virus attenuation (31). It is therefore possible for us to adjust the level of attenuation of the NAGT mutant by using this strategy or other approaches.

Healthy individuals are regularly exposed to α-Gal epitopes via exogenous sources (e.g., food). These regular exposures also explain why different forms of α-Gal antibodies, including serum IgG, are maintained at high levels in healthy individuals. Although it is unlikely, we cannot exclude the possibility that repeated immunizations with the NAGT mutant might cause undesirable side effects in healthy individuals. Further work on the safety aspect of this vaccine approach is warranted.

The use of *in vitro* reactions to enzymatically label antigens with α-Gal epitopes have been used for making experimental inactivated vaccines. We believe that the use of the NAGT mutant as a live attenuated influenza virus vaccine (LAIV) has several additional advantages. First, the expression of α-Gal epitopes by infected cells can obviate the above-mentioned enzymatic labeling and its downstream purification steps. Second, the administration of NAGT mutant via respiratory route can induce mucosal immune responses. Third, the extent of NAGT virus replication in humans *in vivo* can be safely controlled by the naturally expressed anti-α-Gal antibody. Fourth, infected cells labeled with anti-α-Gal antibody can trigger antibody-mediated cellular immune responses. Finally, multiple viral antigens expressed by infected cells can be more readily be taken up by professional APCs for antigen presentation, thereby enhancing downstream vaccine-induced responses against a wide spectrum of viral proteins.

## MATERIALS AND METHODS

### Generation of the NAGT mutant.

The mouse α-1,3-GT gene was amplified via reverse transcription-PCR from spleen tissues of a BALB/c mouse and introduced into a plasmid expressing PR8 NA vRNA (pHW2000-PR8-NA) (32), as indicated in Fig. 1. Recombinant PR8 WT and NAGT viruses were generated by standard reverse genetic technologies (32). Rescued viruses were cultured in 10-day-old embryonated chicken eggs for further amplification. Viral sequences of rescued viruses were confirmed by standard Sanger sequencing.

### Western blotting analyses.

Infected A549 cells (multiplicity of infection [MOI] = 1) were harvested at 24 h postinfection. Cell lysates were resolved in 12% resolving polyacrylamide electrophoresis gels. Rabbit anti-neuraminidase antibody (1,000× diluted; ab21304; Abcam) and horseradish peroxidase (HRP; 2,000× diluted) goat anti-rabbit IgG (ab205718; Abcam) were used as the primary and secondary antibodies, respectively, in the assay.

### Immunofluorescence staining assays.

Infected cells (MOI = 1) were fixed in 4% paraformaldehyde solution in phosphate-buffered saline (PBS) at 24 h postinfection as described previously (33). Mouse anti-α-Gal monoclonal IgM (5× diluted; M86; Enzo Life Sciences) and fluorescent-dye-conjugated goat anti-mouse IgG/IgA/IgM antibody (2,000× diluted; 10667; Invitrogen) were used as the primary and secondary antibodies, respectively, in the assay. DAPI (4′,6-diamidino-2-phenylindole; Invitrogen) was used as a nuclear counterstain.

### Viral replication kinetics assays.

MDCK cells were infected with influenza virus (MOI = 0.001) in triplicate. Infected cells were briefly washed with acidified PBS (pH 2.0) once and PBS (pH 7.0) twice after a 1-h virus adsorption period at 37°C, followed by supplementing with virus culture medium as described previously (33). The titers of progeny virus were determined by standard plaque assays.

### Antibody-dependent NK cell assays.

Human A549 cells were infected with NAGT or WT PR8 virus at an MOI of 10 for 5 h as described previously (34). Infected cells were stained with phycoerythrin (PE)-conjugated Cy7 anti-human HLA-A, -B, and -C antibody (clone W6/32; Biolegend). PBS-treated cells were used as a negative control. Treated A549 cells were incubated with anti-human CD107a–allophycocyanin (clone LAMP-1; Biolegend)-treated CD16.NK92 cells (Fox Chase Cancer Centre) in the presence of heat-inactivated human serum (1:20 dilution) for 5 h. After the incubation, cell mixtures were stained by anti-human CD56–PE antibody (clone MEM188; Biolegend) and fixed. Treated cell mixtures were then stained with anti-NP-fluorescein isothiocyanate (FITC; Abcam) antibodies. Signals for NK cell activation (CD56^+^ CD107a^+^) and those for infected A549 cells (NP^+^ HLA^+^) were acquired with a BD LSR Fortessa cytometer. Signals generated by infected cells without preincubation with human serum and those from mock-infected cells with and without preincubation with human serum were used as controls. All data were analyzed using FlowJo. The percent reduction in infected cells due to α-Gal expression was calculated as follows:

$$\frac{\left(\text{\% of NP}+\text{HLA}+\text{ cells in the presence of serum}\right)-\left(\text{\% of NP}+\text{HLA}+\text{ cells in the absence of serum}\right)}{\text{\% NP}+\text{HLA}+\text{ cells in the absence of plasma}}\times 100\text{\%}$$

### ADCC.

A commercial luciferase reporter assay kit was used for measuring ADCC activities (ADCC receptor bioassay kit; Promega). In brief, A549 cells were infected with WT or NAGT mutant (MOI of 10). At 5 h postinfection, cells were coincubated with diluted human serum and recombinant Jurkat effector cells for 6 h. Cell mixtures without the addition of human serum were used as controls. Luciferase activities of effector cells induced by infected cells were measured by standard luciferase assays. The induction (fold) of ADCC response was calculated as follows:

$$\frac{\text{relative luminometer units from reaction in the presence of human serum}}{\text{relative luminometer units from reaction in the absence of human serum}}$$

### α-1,3-GT KO mouse model, immunization, and virus challenge studies.

WT C57BL/6 and its α-1,3-GT KO mice (11) were purchased from Shanghai Model Organisms Center, Inc., China. To induce anti-α-Gal antibody in KO mice, mice were intraperitoneally injected with rabbit RBC membranes (3 × 10^8^ in 100 μl PBS) at 4 and 6 weeks of age (12). Serum from treated mice was collected at 4, 6, and 8 weeks of age. Serum anti-α-Gal IgG1 antibody levels in treated mice were measured by enzyme-linked immunosorbent assay (ELISA) (see below). For vaccination, NAGT virus (150 PFU in 25 μl PBS) was given intranasally to KO and WT mice at 8 weeks of age. Mock-vaccinated (PBS) WT and KO mice were used as controls.

Treated mice were anesthetized and challenged intranasally by a lethal dose (H1, 40 MLD_50_s; H3 or H5, 10 MLD_50_s) of influenza virus 3 weeks after vaccination as described previously (14). Influenza viruses used in this study were PR8 (A/Puerto Rico/8/1934; H1N1), mouse-adapted HK68 (A/Hong Kong/1/68; H3N2), and highly pathogenic avian H5N1 (A/Vietnam/1203/2004; H5N1) viruses. Morbidity and weight loss were monitored for 14 days, and survival curves were recorded. A weight loss of 30% was set as the humane endpoint for euthanasia. Tissue samples from representative mice were collected at various time points as specified in Fig. 2. All H5 studies were performed in biosafety level 3 facilities.

All animal experiments were approved by the Committee on the Use of Live Animals in Teaching and Research (CULATR) of the University of Hong Kong.

### Phagocytosis assays.

Human monocyte-derived macrophages were prepared as described previously (35). The day prior to the experiment, A549 cells were infected with WT or NAGT virus (MOI = 1), followed by an overnight culture. Infected cells were incubated with human serum on the day of experiment at 4°C for 30 min. Serum-treated cells were added to the macrophages in a 3:1 ratio, and the cell mixture was incubated at 37°C for 2 h. Incubated cell mixtures were washed with PBS, fixed in 4% paraformaldehyde, and then stained with Giemsa stain. Stained macrophages were examined under a standard light microscope. Procedures for collecting primary human monocytes and human serum samples were approved by a local institutional review board.

### Viral load assays.

Lungs from challenged mice were harvested at days 3 and 7 postinfection (*n* ≥ 3), followed by mechanical homogenization. The virus loads were titrated by assays to determine 50% tissue culture infective doses in MDCK cells using the Reed-Muench formula.

### Serological assays.

Heat-treated serum samples from before and after virus challenge were studied by ELISA and microneutralization (MN) assay to measure influenza virus-specific antibody titers, as described elsewhere (14, 16).

For ELISA, Galα1-3Galβ1-4GlcNAc-BSA (bovine serum albumin; 3-atom spacer; Dextra) with recombinant NP or HA (Sino Biological) was used to coat 96-well ELISA plates (Nunc MaxiSorp) at 4°C overnight. Pooled serum samples (≥4 per group) were 2-fold serially diluted, starting from 1:40. HRP-conjugated anti-mouse IgG1 antibody (31430; Invitrogen) was used as the secondary antibody. Absorbance at 450 nm was measured. Each sample was tested in duplicate. A ≥4-fold difference in endpoint titer was considered significant.

For MN assays, pooled receptor-destroying enzyme (RDE)-treated serum samples (≥ 3 per group) were 2-fold serially diluted, starting from 1:10, and tested against PR8 or HK68 virus. The neutralization antibody titers were expressed as geometric mean titers.

### Cell profiling in infected samples.

Mouse lung and spleen tissues were harvested at days 1, 3, 5, and 21 postvaccination, as well as days 3 and 7 postinfection (*n* ≥ 5) using the protocol described previously (14). After hemolysis with RBC lysis buffer (eBioscience), cells were stained with Zombie Aqua (Biolegend) and then stained for different cell markers (Fc block by anti-CD16/CD32 [BD Biosciences] and anti-CD11b–FITC, anti-CD11c–perdinin chlorophyll protein [PerCP]-Cy5.5, anti-Ly6G–allophycocyanin, anti-F4/80–PE, and anti-MHC II-PB [Biolegend]). Stained cells were fixed (BD Cytofix/Cytoperm buffer) as described previously (14). Markers for DCs (CD11c^+^ MHCII^hi^ Ly6G^−^ F4/80^−^), neutrophils (Ly6G^hi^ CD11b^hi^), alveolar macrophages (CD11c^+^ F4/80^+^) in lungs, and macrophages (CD11b^+^ F4/80^+^) in spleens were studied. Signal acquisition was performed on a BD LSR Fortessa cytometer, and data were analyzed with FlowJo. Gating strategies for these cells are shown in the figures.

### ICS assays.

CD4^+^ and CD8^+^ T-cell recall responses were determined as described previously (36, 37). The production of type 1 (IFN-γ, TNF-α, and interleukin 2 [IL-2]) and type 2 (IL-4) cytokines of T cells was detected. Splenocytes and cells from BAL fluid (*n* ≥ 3 fluid samples per group) were harvested at day 7 postinfection. After RBC lysis, isolated lymphocytes were stimulated by PR8 or HK68 virus and coincubated with anti-CD28, anti-CD49d (BD Biosciences), and IL-2 (Roche) for 6 h, followed by an incubation with GolgiPlug (containing brefeldin A; BD Biosciences) overnight. For splenocytes at 3 weeks after vaccination, PR8, HK68, H1N1/Brisbane/07 (H1N1), and HK/MPF461/07 (H5N2) viruses were used to stimulate the cells. Treated cells were first stained with Zombie Aqua (Biolegend) and then with T-cell markers (Fc block by anti-CD16/CD32 [BD Biosciences]; anti-CD4–allophycocyanin/Cy7 and anti-CD8–PerCP/Cy5.5 [Biolegend]). Stained cells were then fixed with Cytofix/Cytoperm buffer (BD), followed by intracellular cytokine staining (IFN-γ, FITC; TNF-α, allophycocyanin; IL-2, PE; and IL-4, PE/Cy7; Biolegend). Signal acquisition was performed on a BD LSR Fortessa cytometer, and data were analyzed with FlowJo.

### Statistical analyses.

Unless stated otherwise, one-way analysis of variance was used to determine the effect on the magnitude of cellular immune responses, cell population profiles, and viral loads within WT and KO groups, while the *t* test was used for the same purpose to compare WT and KO mice at the same time point. Survival curves based on weight loss were analyzed with GraphPad Prism. The log rank test was used to compare the survival rates between groups.
