# Supplementary material for: Heterosubtypic Protection Induced by a Live Attenuated Influenza Virus Vaccine Expressing Galactose-α-1,3-Galactose Epitopes in Infected Cells
Source: mBio. 2020 Mar 3;11(2):e00027-20. doi: 10.1128/mBio.00027-20 (PMC7064743; doi:10.1128/mBio.00027-20)
Supplement: FIG S1 [file mBio.00027-20-sf001.pdf]

Yan et al. Fig S1

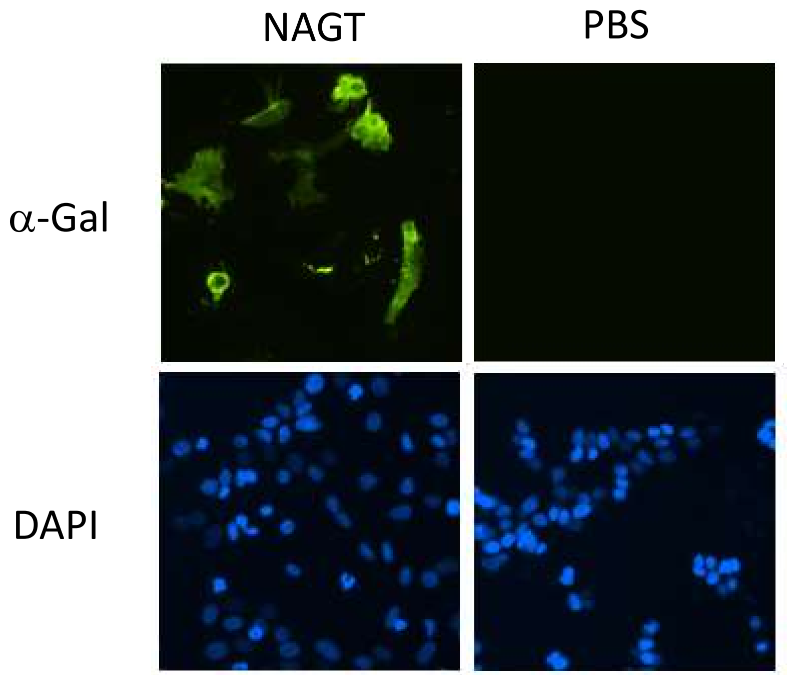

Figure S1. Detection of α-Gal epitopes on non-permeabilized cells. Human A549 cell treated with NAGT mutant and PBS (control) were stained for α-Gal epitopes. The staining protocol is identical to the one used in Fig. 1D, except Triton X-100 was not used in the initial cell fixation. DAPI was used as a nuclear counterstain.
