# Supplementary material for: Heterosubtypic Protection Induced by a Live Attenuated Influenza Virus Vaccine Expressing Galactose-α-1,3-Galactose Epitopes in Infected Cells
Source: mBio. 2020 Mar 3;11(2):e00027-20. doi: 10.1128/mBio.00027-20 (PMC7064743; doi:10.1128/mBio.00027-20)
Supplement: FIG S2 [file mBio.00027-20-sf002.pdf]

Yan et al. Fig S2

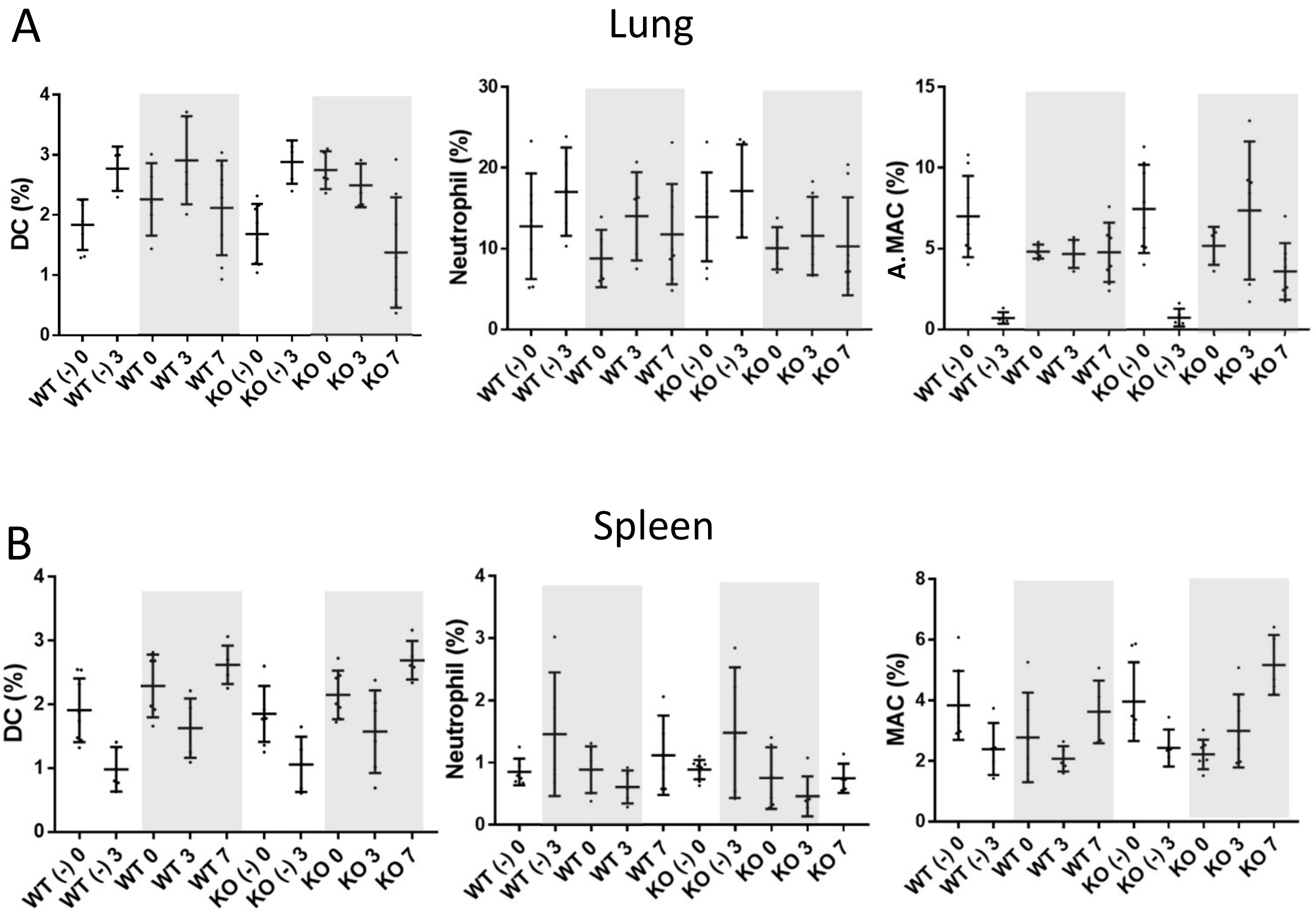

Figure S2. NAGT mutant protects mice from a lethal homologous virus challenge. 3 weeks after vaccination, mice were challenged (i.n) with a lethal dose of H1N1 (PR8;10MLD$_{50}$). (A) Percentages of total dendritic cells, neutrophils and alveolar macrophages in lung tissues (left to right). (B) Percentages total dendritic cells, neutrophils and macrophages in spleen tissues (left to right). Data represent Mean ± SD.
