# Supplementary material for: Heterosubtypic Protection Induced by a Live Attenuated Influenza Virus Vaccine Expressing Galactose-α-1,3-Galactose Epitopes in Infected Cells
Source: mBio. 2020 Mar 3;11(2):e00027-20. doi: 10.1128/mBio.00027-20 (PMC7064743; doi:10.1128/mBio.00027-20)
Supplement: FIG S3 [file mBio.00027-20-sf003.pdf]

Yan et al. Fig S3

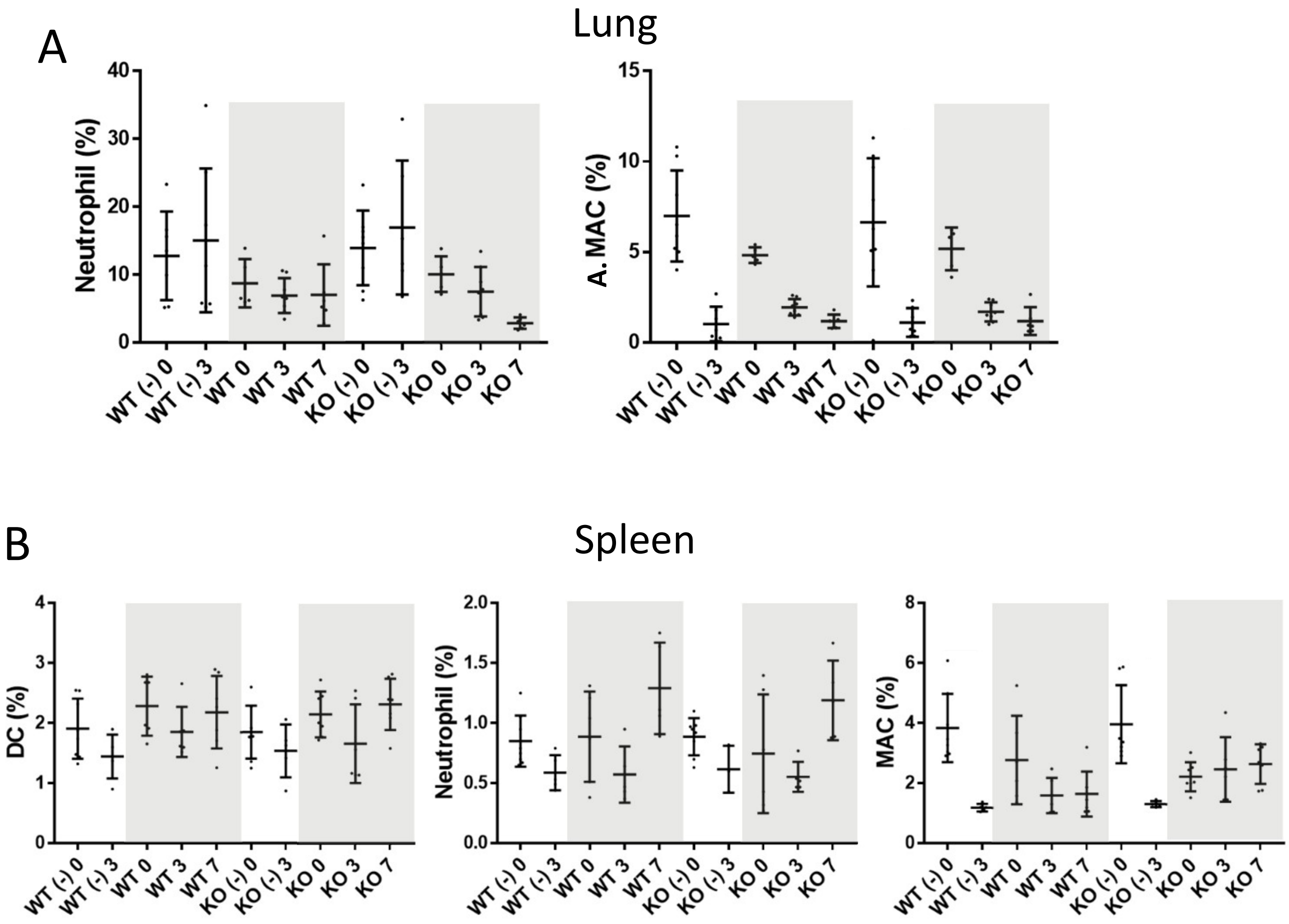

Figure S3. NAGT mutant protects mice from a lethal heterosubtypic H3N2 virus challenge. 3 weeks after vaccination, mice were challenged (i.n) with a $10MLD_{50}$ dose of H3N2/HK68. (A) Percentages of neutrophils (left) and alveolar macrophages (right) in lung tissues. (B) Percentages total dendritic cells, neutrophils, and macrophages in spleen tissues (left to right). Data represent Mean ± SD.
