# Supplementary material for: Heterosubtypic Protection Induced by a Live Attenuated Influenza Virus Vaccine Expressing Galactose-α-1,3-Galactose Epitopes in Infected Cells
Source: mBio. 2020 Mar 3;11(2):e00027-20. doi: 10.1128/mBio.00027-20 (PMC7064743; doi:10.1128/mBio.00027-20)
Supplement: FIG S4 [file mBio.00027-20-sf004.pdf]

Yan et al. Fig S4

A) H1N1/PR8

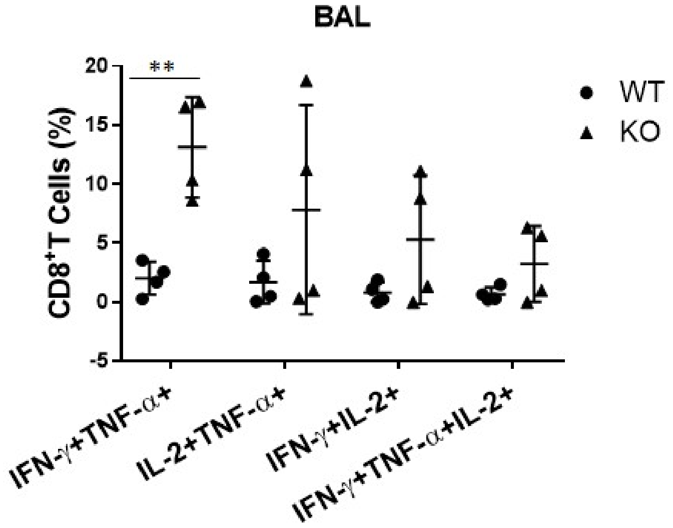

B) H3N2/PR8

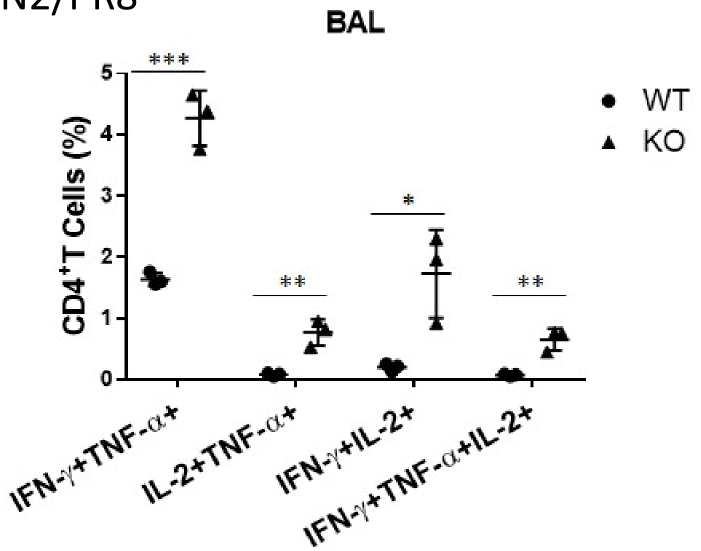

Figure S4. NAGT mutant vaccination can enhance polyfunctional T cell responses in BAL upon virus challenge. A) PR8-specific polyfunctional $CD8^+$ T cells in BAL at day 7 post-challenge. Data are re-analysed from those presented in Fig. 5E (right panel). B) HK68-specific polyfunctional CD4+ T cells in BAL at day 7 post-challenge. Data are re-analysed from those presented in Fig. 6F (left panel) Data represent Mean ± SD; *: $p < 0.05$, **: $p < 0.01$, ***: $p < 0.001$.
